# Clinical and Electrophysiological Characterization of Essential Tremor in 18 Children and Adolescents

**DOI:** 10.5334/tohm.803

**Published:** 2023-12-20

**Authors:** Julie Piarroux, Evgenia Dimopoulou, Guillaume Taieb, Sarah Souvannanorath, Emmanuel Roze, Laurence Lion-François, Marie-Aude Spitz, Emmanuel Broussolle, Chloé Laurencin, Jean-Baptiste Chanson, Johanna Belleville-Goffeney, Marie Céline François-Heude, Pierre Meyer, Mirna Khalil, Maelle Dereure, Diane Doummar, Hugues Chevassus, Emmanuelle Apartis, Agathe Roubertie

**Affiliations:** 1 Department of Pediatric Neurology, Gui de Chauliac University Hospital, Montpellier, France; 2 University of Montpellier, CNRS (IGF), Department of Neurology, Montpellier, University Hospital 34295 Montpellier, France; 3 Reference center for neuromuscular diseases, Henri-Mondor University Hospital, Assistance publique-Hôpitaux de Paris, Créteil, France; 4 Department of Neurology, Salpêtrière Hospital, Sorbonne University and Assistance Publique - Hôpitaux de Paris, Paris, France; 5 Department of Pediatric Neurology, Lyon University Hospital, Bron, France; 6 Department of Pediatry, Strasbourg University Hospitals, Strasbourg, France; 7 Research Unit UMR 5229, Marc-Jeannerod Institute of Cognitive Science, French National Center for Scientific Research (CNRS), University of Lyon, Bron, France; 8 Department of Neurology C, Civil Hospices of Lyon, Pierre-Wertheimer Neurological Hospital, Bron, France; 9 Faculty of Medicine Lyon-Sud Charles-Mérieux, University of Lyon, Oullins, France; 10 Lyon Neuroscience Research Center (CRNL), INSERM U1028, CNRS UMR5292, University Lyon 1, Lyon F-69000, France; 11 Department of Neurology, Strasbourg University Hospitals, Strasbourg, France and Reference centre for neuromuscular diseases Nord/Est/Ile-de-France, France; 12 Departement of Pediatric Neurology, Jean-Minjoz University Hospital, Besançon, France; 13 Clinical Investigation Center, Montpellier University Hospital, France. INSERM, CIC1411, Montpellier, France; 14 Clinical Research and Epidemiology Unit, La Colombière University Hospital, Montpellier, France; 15 Department of Pediatric Neurology and developmental pathologies, Sorbonne University and Trousseau University Hospital, Assistance Publique-Hôpitaux de Paris, Paris, France, APHP. SU, FHU I2D2, F-75012, Paris, France; 16 Department of Neurophysiology, Saint-Antoine Hospital, Assistance Publique-Hôpitaux de Paris, INSERM U1127, CNRS UMR7225, UM75, ICM, 75013 Paris, France; 17 Institute for Neurosciences of Montpellier, INSERM U 1298, University of Montpellier, Montpellier, France

**Keywords:** essential tremor, children, quality of life, myoclonic jerks

## Abstract

**Background:**

Essential tremor (ET) is considered the most frequent abnormal movement in the general population, with childhood onset in 5 to 30% of the patients.

**Methods:**

A multicenter, descriptive cross-sectional study enrolled patients ⩽18 years with a definite diagnosis of ET according to the International Parkinson and Movement Disorders Society criteria. Demographic data, clinical and electrophysiological characteristics of the tremor, neurological examination and impact on quality of life were collected.

**Results:**

9 males and 9 females were included (mean age of 13.9 years). Tremor was characterized by : upper limb onset at a mean age of 6.5 years; at enrollment, upper limbs localization, and involvement of an additional body region in 28% of the patients; kinetic tremor in all of the patients combined with postural tremor in 17 and rest tremor in 3; tremor mean frequency of 7.6 Hz, mean burst duration of 82.7 ms; identification of mild myoclonic jerks on the polymyographic recordings in 7 patients; altered quality of life with worse emotional outcomes in girls and when a disease duration >5 years was suggested.

**Discussion:**

Childhood-onset ET is associated with delayed diagnosis and remarkable functional impact. Electromyographic identification of additional mild myoclonus is a new finding whose significance is discussed.

**Highlights:**

ET onset involved upper limbs and at inclusion, 28% of the patients exhibited involvement of an additional body region.

ET impacted quality of life for all patients.

Girls and patients affected for >5 years reported worse emotional outcomes.

Mild myoclonic jerks were identified on 7/17 polymyographic recordings.

## Introduction

The International Parkinson and Movement Disorders Society (MDS) defines essential tremor (ET) as *an isolated tremor syndrome of bilateral upper limb postural or kinetic tremor, with or without head tremor or tremor in other locations*, with at least a 3-years history of tremor and no neurological cause found [1]. ET more typically affects the upper limbs (UL) on a bilateral, sometimes asymmetrical, mode. The natural history of ET in adults is usually one of a progressive worsening and gradual expansion of the tremor [2]. The diagnosis of ET is complex, especially since it can be combined with neurodegenerative disorders (notably Parkinson’s diseases) [2,3,4]. In adults, ET is associated with an overall decrease in quality of life, mainly in the psychosocial domain [5].

ET is usually considered the most frequent abnormal movement in the general population [6]. Its prevalence ranges between 0.4% and 4% of the adult population [6] and increases with aging up to 14.3% of people over 60 [6]. However, several studies have shown that the first symptoms can appear much earlier in life [7,8]. The age at onset follows a bimodal curve, with a peak in late childhood and another around 60 [3,9]. Adult-populations based studies have shown an onset in childhood in 5% to 30% of cases [7,10,11].

The mean age of childhood-onset essential tremor (COET) is estimated to be around 6 years [12]; cases of very early onset have been described, including neonatal presentations [13]. Very few clinical and electrophysiological studies have been performed in children, all retrospective cohorts or very small groups of patients or studies in adult patients with childhood onset, and the functional impact has not been systematically evaluated [7,10,12,13,14,15,16].

The aim of this study is to carry out a precise clinical and electrophysiological characterization of COET and to assess its functional impact.

## Material and methods

### Patients

This cross-sectional study enrolled patients from five university hospitals in France between July 2018 and March 2021.

Patients who fulfilled the following criteria were included:

– age between 3 and 18 years at assessment;
– diagnosis of essential tremor established according to the diagnosis criteria of the MDS (1998) [17], by a pediatric neurologist or a neurologist specialized in movement disorders;
– normal cerebral MRI in patients without any family history of ET;
– normal thyroid function.

Patients were recruited prospectively or retrospectively. Retrospective enrollment was possible if the patient (i) fulfilled the above inclusion criteria (ii) benefited previously of clinical examination and polymyographic recording as daily care, and before the age of 18; the age at this evaluation was considered as the age at assessment.

All patients with a potentially intercurrent neurological condition were excluded (conditions listed in supplementary Table 1); other causes of tremor, especially dystonic tremor and/or myoclonus dystonia, were carefully investigated by clinical and electrophysiological assessment, and patients presenting with those pathology were not included in our study. Genetic testing was not part of the study design as (i) MDS criteria for ET diagnosis does not include genetic criteria, (ii) differential diagnoses especially of dystonic tremor and myoclonic dystonia rely on clinical assessment and not genetic testing, (iii) genetic bases of ET are not definite.

### Clinical evaluation

Demographic characteristics, family history of ET, perinatal history and psychomotor development, age at onset, age at diagnosis, characteristics of the tremor (location, activation conditions, alleviating or worsening factors, and evolution) and effects of medications were collected in the medical records and by familial interview (treatment effect was ascertained by patients’ subjective impression as marked, moderate, mild or no improvement).

Clinical assessment was performed by one single physician expert in movement disorders in each center (LLF, MAS, ER, DD, AR), and included general and neurological examination focused on the characteristics of the tremor (location, activation conditions, drawing of Archimedean spiral, test of the ladder rung, writing exercise, following a standardized protocol). Data concerning psychological, social and academic repercussions of ET were collected by family interview and quality of life was rated by the patient and parent or proxy using the Pediatric Quality of Life inventory (PedsQL [18]).

The data of retrospectively enrolled patients were collected from the medical file. Only data from the last visit with examination by the physician expert in movement disorders were considered.

### Polymyographic evaluation (PMG)

A four-way polymyographic recording of surface electromyogram (PMG) coupled with accelerometry [14] was performed by one single physician trained in pediatric tremor recording in each center (JBC, CL, JP, GT, EA) for prospectively included patients.

**Figure 1:**
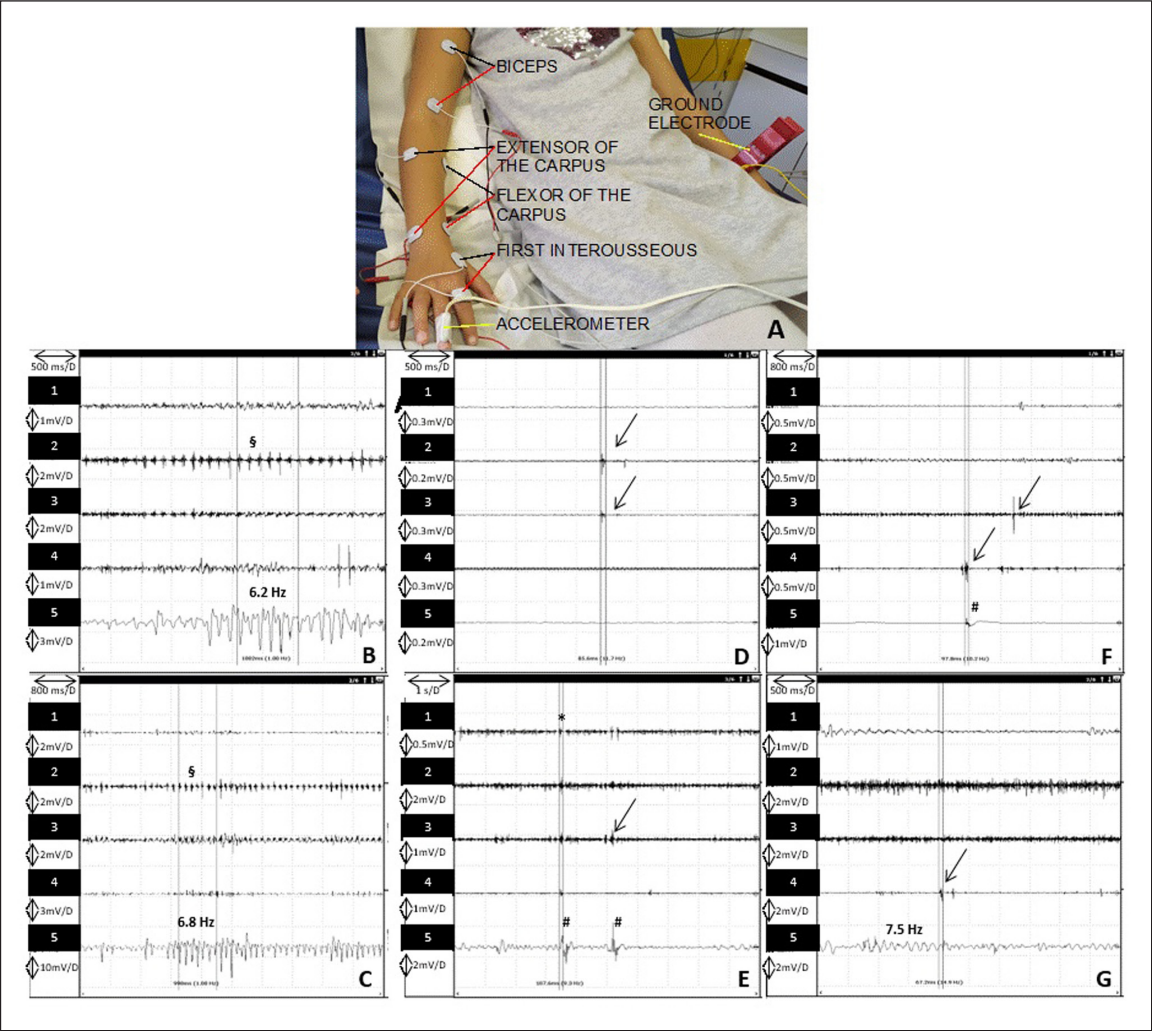
Polymyographic (PMG) recordings. **A:** Installation of the patient for PMG recordings. Recording was performed in the following muscles; flexor and extensor of the carpus, biceps, and first interosseous muscle of the hand. Black lines show the active electrode, red lines the reference electrode associated to the same muscle. Accelerometer and ground electrode (common for all pairs of electrodes) are pointed with a yellow line. **B, C:** PMG recordings of tremor. Trace 1: biceps; Trace 2: extensor carpi radialis (ECR); Trace 3: flexor carpi radialis (FCR); Trace 4: first dorsal interosseous (FDI); Trace 5: accelerometer. **B:** 6.2 Hz regular tremor in a 13 years-old male patient visible on the accelerometer and on the FCR and ECR; arms in the mirror position. **C:** 6.8 Hz tremor in a 16 years-old female patient visible on the accelerometer and on the FCR ; carpus extended. **D-G:** PMG recordings of myoclonus (we selected samples of myoclonus in patients exhibiting tremor (not shown)). **D, E:** myoclonus recordings in a 17 years-old male patient visible at rest (D) and while maintaining the mirror position (E). **F, G:** myoclonus recordings in a 13 years-old male patient (same patient as B tremor recording), at rest (F) and while extending the carpus (G) (note the 7.5 Hz tremor visible on the accelerometer). Black arrows indicate myoclonus on the PMG; #: myoclonus visible on the accelerometer; *: synchronous myoclonus on all muscle tested; $: tremor burst on the PMG correlates with the accelerometer.

The explored limb was the most affected upper limb (greater amplitude of tremor or greatest functional complain) for each child. Recorded muscles are shown on Figure 1. Muscle activity was recorded successively at rest, in posture, during maneuvers such as mental arithmetic or the contralateral repetitive motor test and finally in action.

The addition of a weight during recording was used to differentiate essential tremor from exaggerated physiological tremor, as both conditions can share common frequencies. he frequency usually decreases when loads are applied to the upper limbs during neurophysiological recordings in patients with enhanced physiological tremor, which is not observed in essential tremor.

Frequency regularity of tremor and bursts’ duration were also assessed.

Available PMG data were collected from the medical file of the patients retrospectively included.

### Statistical analyzes

Comparisons between subgroups (sex, disease duration, age at onset, age at assessment, family history of ET) were performed using Student’s t test for quantitative normally distributed data, Wilcoxon-Mann-Whitney test for other quantitative data, and Fisher’s exact test for qualitative data. Pearson’s product-moment correlation was used for correlations. Statistical differences were deemed significant for a p value < 0.05, adjustment for false discovery rate (FDR) was performed using the R package fdrtool [19]. The analyses were made using SAS Enterprise Guide, version 4.3 (SAS Institute, Cary, NC, USA).

### Ethical and regulatory considerations

This study was sponsored by Montpellier University Hospital (France, PROM9770). It was approved by the French Institutional Ethics Committee “*Comité de Protection des Personnes Ile de France 8*” (number 1707–44), and carried out in accordance with the Declaration of Helsinki, International Conference on Harmonization guidelines for Good Clinical Practice and French regulatory requirements. The study was registered on the U.S. clinical trial database “ClinicalTrial.gov” before the first patient inclusion (NCT03487705). All patients and their caregivers gave written informed consent to the research and to publication of the results.

## Results

### Patients

**Figure 2:**
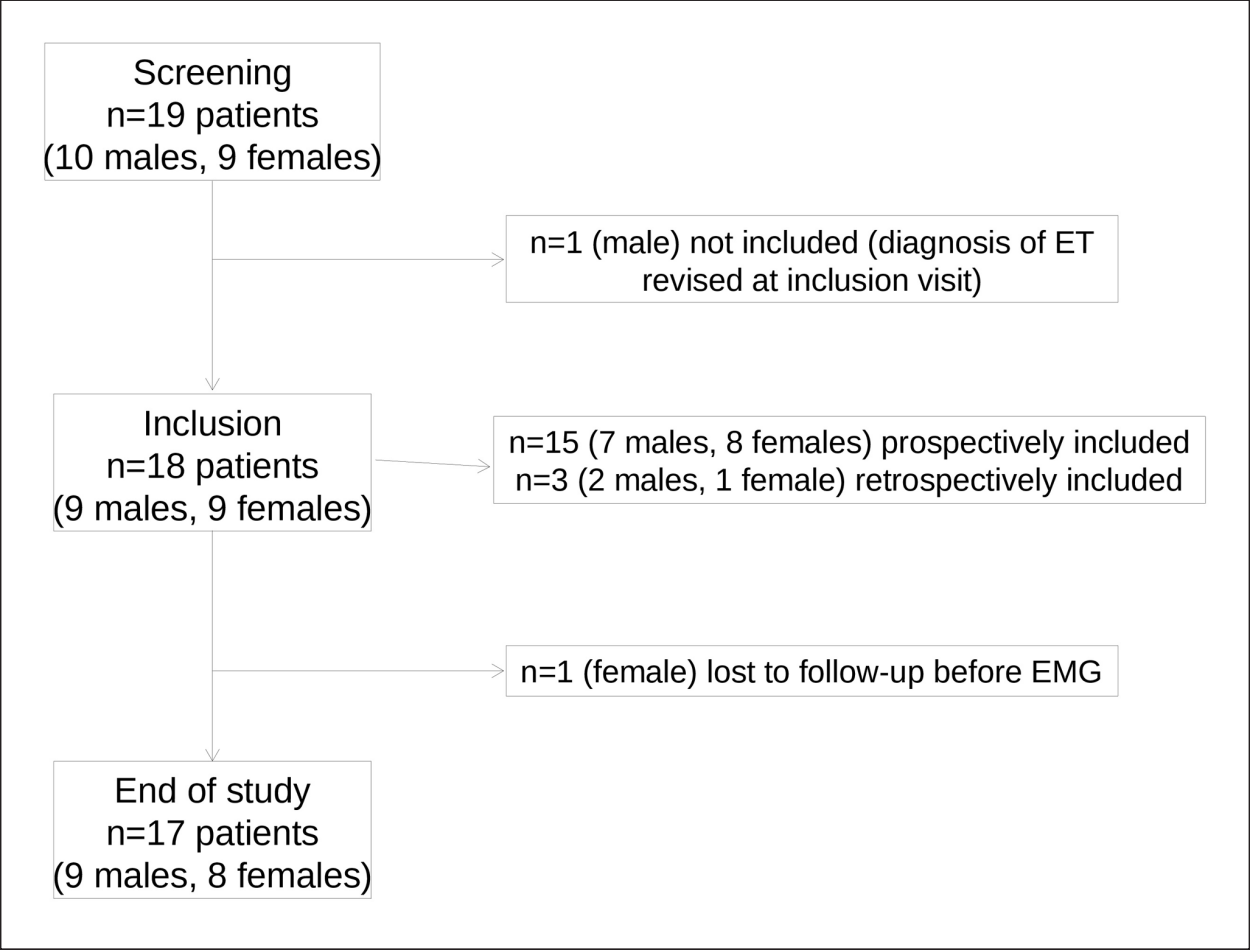
Flow chart. n = number of patients.

Nineteen patients were screened and 18 were enrolled in the study (9 males and 9 females), including 3 retrospectively; 17 completed the study (Figure 2). One patient didn’t fill-in the PedsQL Parent and Self-Report, and two didn’t answer the PedsQL Self-Report. Patients’ characteristics are described in Table 1. All PMG were recorded between the age 8.1 and 17.4. Diagnosis delay ranged from 5 to 130 months (median 30 months). All patients had normal development and neurological examination. A family history of ET was reported for 11 patients. There was no significant difference in family history between boys and girls or depending on the age at onset.

**Table 1:** Main characteristics of the patients, according to sex.


	MALE	FEMALE	TOTAL

NUMBER OF PATIENTS	9	9	18

Age at inclusion^1^ (years)	14.6 ± 5 [8–24]	3.2 ± 4.1 [8–20]	13.9 ± 4.7 [8–24]

Age at onset^2^ (years)	7.1 ± 4.43 [2–13]	5.9 ± 2.30 [2–10]	6.5 ± 3.5 [2–13]

Age at diagnosis (years)	11.5 ± 4.3 [3.8–15.4]	9.2 ± 3.5 [3.6–14.7]	10.3 ± 4 [3.56–15.4]

Disease duration at inclusion (years)	7.6 ± 6.3 [2.84–19.6]	7.2 ± 3.8 [2.3–14.6]	7.4 ± 5.1 [2.3–19.6]

Hand lateralization^3^: right	88% (7)	100% (8)	94% (15)

Normal perinatal history	100% (9)	100% (9)^4^	100% (18)

Sleep disturbances	22% (2)	14% (1)	23% (3)

Attention difficulties according to the family	22% (2)	0% (0)	13% (2)

Other problem^5^	11% (1)	0% (0)	6% (1)

Family history of ET^5^	56% (5)	67 (6)	61% (11)

Parent affected: mother	(1)	(2)	(3)

father	(3)	(1)	(4)

sibling	(0)	(1)	(1)

second degree relative^6^	(2)	(2)	(4)

Personal assistance:	86% (6)	33% (3)	56% (9)

Psychomotor therapist	(2)	(1)	(3)

Computer alone	(1)	(2)	(3)

Occupational therapist + computer	(2)	(0)	(2)

Extra time for tasks	(1)	(0)	(1)

Psychological distress	71% (5)	44% (4)	56% (9)

Psychological follow-up	37% (3)	25% (2)	31% (5)

Personal assistance and/or psychological follow-up	78% (7)	50% (4)	65% (11)

Course of the disease from beginning according to the patient and parents:			

worsened	63% (5)	50% (4)	56% (9)

stable	25% (2)	38% (3)	31% (5)

improved	13% (1)^7^	13% (1)	13% (2)

PedsQL global score (patient)	86.3 ± 6 [77.2–91.3]	73.8 ± 13.1 [52.1– 88]	79 ± 12.2 [52.2–91.3]

PedsQL global score (parent)	79.8 ± 12.6 [54.3–91.3]	75.6 ± 21.3 [33.7–91.3]	77.7 ± 17 [33.7–91.3]


Qualitative data are presented as percentage (number of patients); quantitative data are presented as mean ± standard deviation [range]. 1: For prospective inclusions, age at inclusion ranged from 8 to 17; 2: unknown for one girl; 3: one boy was left handed, no patient was ambidextrous 4: one twin pregnancy with delivery at 37 weeks; 5: dysorthographia; 6: one patient had a father and a grandfather, one had a grandfather and an uncle, the other two had an uncle affected; 7: this patient was treated by propranolol; PedsQL: Pediatric Quality of Life inventory.

### Tremor

#### Clinical characterization

Tremor characteristics at onset, at inclusion according to patient or caregivers’ assessment, and during clinical examination are reported in Tables 2 and 3. An upper limb (UL) tremor was observed in all but one patient, with an asymmetry in 10/18 patients. Four patients also presented with postural lower limbs (LL) tremor and one also had voice tremor. No other tremor localization was reported by the physicians, but four patients reported tremor of other body parts (Table 3).

**Table 2:** Main characteristics of tremor and its course: clinical and electrophysiological evaluation.


	CLINICAL ASSESSMENT:

Upper limb	100% (18)

Lower limb	22% (4)

Voice	6% (1)

Neck	0% (0)

Head	0% (0)

More than one localization	28% (5)

Bilateral Asymmetry	94%^1^ (17) (9)

Postural or action	100% (18)

Postural	94% (17)

Postural alone	0% (0)

Postural + action	7% (1)

Action	94% (17)

Action alone	(1)

Only during a specific task^2^	(1)

Rest tremor	17% (3)

Isolated (number of patients)	(0)

Rest tremor + action tremor	(0)

Rest tremor + action tremor + postural tremor	(3)

Associated with myoclonus	25% (4)

	Electrophysiological assessment:

Postural or action tremor	88%^3^ (15)

Rest tremor	6% (1)

Associated with myoclonus	41% (7)

Mean frequency of tremor (Hz)	7.6 ± 1.4 [5–10]

Mean duration of bursts (ms)	82.7 ± 16.0 [65–130]

Weight (500g) persistence^3^	93% (13)

Mean frequency for tremor persisting with 500 g weight (Hz)	8.18 ± 1.47 [4–10]


Qualitative data are presented as percentage (number of patients); quantitative data are presented as mean ±standard deviation [range]. 1: for one boy, only left upper limb (UL) tremor was observed during the evaluation, although he reported having intermittent tremor of the right UL; 2 : while pouring water; 3: tremor was very subtle during EMG assessment in 2 patients and could not be recorded.

**Table 3:** Main characteristics of tremor and its course: course of disease according to patients and/or parents/caregivers.


	AT ONSET	AT INCLUSION

Topography (body distribution)		

Upper limb	100% (18)	100% (17)

Lower limb	0% (0)	35% (6)

Voice	6% (1)	23% (4)

Neck	0% (0)	6% (1)

Head	0% (0)	23% (4)

More than one localization	6% (1)	47% (8)

Bilateral	100% (18)	100% (17)


Qualitative data are presented as percentage (number of patients).

Action tremor was identified in all (18) patients; 17 patients also showed a postural tremor and three a rest tremor.

Clinical assessment revealed myoclonic jerks associated with tremor in four patients.

All the patients with available data reported at least one worsening factor (16/16), especially stress, which was the most commonly reported (15/15). Only 5/16 patients reported more than 2 worsening factors (supplementary Table 2). The reported alleviating or worsening factors remained stable along the course of the disease for a given patient.

#### Course of disease

All patients had UL tremor from onset. One described concomitant onset of UL and voice tremor. Seven patients reported secondary involvement of at least another body part, confirmed by physical examination in four cases.

Nine patients among the 16 with available information noted a progressive worsening of the tremor from onset. Two described an improvement, one of whom was treated with propranolol.

#### Electrophysiological characterization

Tremor characteristics recorded in 15 patients are reported in Table 2. Action tremor was recorded in all the patients. Among them, two were able to suppress the tremor when concentrating on contralateral ballistic movement; the tremor was never driven by a rhythmic contralateral beat (14 patients tested). A regularity of the tremor was noted for six patients, and the tremor was qualified “irregular” for nine patients. The frequency was 7.6 ± 1.4 Hz [5–10]. There was no difference in tremor frequency between boys and girls, nor according to age of onset, age at examination, duration of disease or the presence of family history (data not shown). Mean duration of burst was 82.7 ± 16 [65–130] ms; these data were not recorded for retrospectively included patients.

Seven patients had myoclonus associated to tremor on the EMG recordings, amongst whom the four patients exhibiting myoclonus at examination. Myoclonus was rare, characterized by isolated brief bursts of activity (34 to 160 ms) on one or a few muscles, most often distal, of low amplitude, mainly observed at rest, but sometimes superimposed with the postural tremor, and only seldom translated into a movement on the accelerometer.

Tremor was very subtle at the time of neurophysiological assessment in two patients and could not be recorded.

### Impact on quality of life and function

All patients reported repercussions of their tremor on autonomy in daily life activity and/or quality of life. Nine children benefited from pedagogical specific measures (Table 1); all the enrolled patients achieved an academic level concordant with their age.

Among the 16 patients with available data, nine complained of psychological distress; a psychological follow-up was required in 5/16 patients.

The PedsQL lowest scores were consistently reported in the emotional functioning scales according to both parents and children assessment (respectively 64.56 ± 20.3).

### Treatment

A treatment was proposed to eight patients, but only five accepted a medication. The first line of therapy was always propranolol (efficient in 3/5 (marked improvement in the three of them, who were still treated at last follow-up, after respectively 5, 3.8 and 1.7 years, daily dose ranging between 60 and 80 mg). The two other patients reported no effect (dailydaily daily dose 20 and 160 mg respectively) and discontinued the treatment after respectively 3 months and 2 years; they received a second line of treatment with primidone. The first one reported no effect of primidone 50mg per day; he discontinued the treatment after 3 months and had a third line of treatment with alprazolam (1mg per day) during 1 month, with no reported effect. The second patient who received primidone (125 mg per day) reported dizziness at the initiation of treatment, which disappeared after some time, and an initial moderate improvement on the tremor; the treatment was discontinued after 2 years due to a loss of effect and the apparition of cognitive slowing.

### Analyses by groups

Emotional score on the self-filled PedsQL charts was lower for girls (53.6 ± 7.5 [25–75]) than boys (80 ± 14.6 [60–95], p = 0.02. This score was also significantly lower for children with a disease duration of more than five years (53.6 ± 16.5 [25–75]) when compared to children with a disease duration of less than five years (80 ± 14.6 [60–95], p = 0.02). After FDR adjustment, these differences lost significance. No other difference could be evidenced between any PedsQL subscores or PedsQL global score in any subgroups of patients (supplementary Table 3).

No difference could be evidenced between patients demographic or clinical characteristics regarding (i) clinical tremor characteristics or (ii) the electrophysiological tremor characteristics, especially regarding age at onset, age at assessment, or sex. Myoclonic jerks were not associated with patients’ or tremor’s characteristics. No difference was identified between patients with or without family history of ET (supplementary Table 4).

## Discussion and conclusion

The main findings of this study are (i) an original description of clinical features and electrophysiological characteristics of ET in children by standardized polymyography; (ii) recording of mild myoclonic jerks in 7 patients, which represents a totally new result; (iii) recognition of a noteworthy impact in daily life activity and/or significant altered quality of life with worse emotional outcomes when a disease duration > five years and in girls; approach of the impact of ET in childhood using dedicated and validated scales has not been performed previously.

According to parental questionnaire, we ascertained that ET onset involved both upper limbs in 100% of the patients. Age at onset (mean age 7 years), and family history of ET in more than half of the patients are concordant with previous studies [7,10,12,13,14,15,16] (Table 4). Mean age at diagnosis (10.3 years) is noteworthy as it has not been specifically analyzed in other series; this data is concordant with diagnosis delay described in most of childhood-onset movement disorders [20].

**Table 4:** Results of the studies of childhood-onset essential tremor.


	PRESENT STUDY	GHOSH *ET AL*. (13)	FUSCO *ET AL*. (14)	LOUIS *ET AL*. (12)	TAN *ET AL*. (7)	JANKOVIC *ET AL*. (8)	LOUIS *ET AL*. (16)

STUDY TYPE (YEARS OF STUDY)	CROSS-SECTIONAL (2018–2021)	RETROSPECTIVE (2004–2011, PUBLISHED IN 2016)	CROSS-SECTIONAL (PUBLISHED IN 2003)	RETROSPECTIVE (PUBLISHED IN 2001)	PROSPECTIVE, CROSS-SECTIONAL (PUBLISHED IN 2006)	RETROSPECTIVE (PUBLISHED IN 2004)	RETROSPECTIVE (PUBLISHED IN 2005)

Number of patients	19	211	9	19	19	39	95

Age at onset^1^	7.1 [2–13]	9.71 ± 5.62^2^	[2–5]	6.8 [1–14]	10.8 ± 4.1 [6–16]	8.8 ± 5.0 [1–16]	5.9 ± 4.7 [1–16]

Age at assessment^1^	13.9	14.09 ± 5.00^3^	[7.5–16.8]	11.3	25.7 ± 15.0 [16–73]	20.3 ± 14.4 [3.3– 64.4]	11.7 ± 4.6 [2–18]

Mean age at diagnosis^1^	10.3 (±4)	NK	NK	NK	NK	NK	NK

Sex distribution (% of males)	50	61	66	68	73.5	74	74

Presence of a family history of tremor (%)	61	35	44	NK	56.2	79.5	62^4^

Course^5^:							

Progressive	56 (9)	40 (88)	NK	NK	NK	NK	NK

static	31 (5)	45 (95)					

improvement	12 (2)	1 (4)					

NK	1 (3)	11 (25)					

Distribution at onset: bilateral upper limb (%)	100	94	NK	NK	NK	NK	NK

Distribution at last assessment: bilateral (%)	100	100	NK	100	100	NK	NK

Other regions involved^5^	(5)	Yes^6^	11 (1)	5 (1)	10.5 (2)	Yes^6^	NK

Head tremor^5^	0	12	0	11 (1)	10.5	17	3.2

Type of tremor^7^:							

Postural	16	Postural and kinetic^6^	9	15	19	34	NK
	
Kinetic	17		9	18	16	27	
	
Rest	3		0	0	0	3	

Treatment (%)	26	29	11	26.3	63.1	61	24.2

Impairement (%)	100	55	NK	Yes^6^	Yes^6^	NK	NK


Qualitative data are presented as percentage (number of patients) and quantitative data as mean ±standard deviation [range] when available except for the type of tremor, where only number of patient is shown. NK: not known; 1: onset was at birth in 7 children; 2: 2 years was the youngest presentation; 3: first degree parents only were reported ; 4: unknown percentage.

Clinical characterization of the tremor is concordant with previous descriptions: bilateral upper limbs localization, postural or action tremor, associated to rest tremor in 3 patients; various worsening factors, especially emotion and stress (in all the patients who reported worsening factors). Electrophysiological assessment identified a mean tremor frequency of 7.6 ± 1.4 Hz and a mean burst duration of 82.7 ± 16.0 ms, as reported in a single study in 9 children, and as commonly observed in adult patients [14,21]. Fusco *et al*. distinguished 2 groups of patients according to their age: young patients and adolescents; in young patients, but not in adolescents, tremor had a lower frequency that increased when loading the extremity with an additional weight [14]. We did not record such paradoxical response.

One of the main findings of our study is the clinical identification of myoclonic jerks, and the recording of myoclonus on the EMG for seven patients. These electrophysiological events were characterized by brief bursts of activity in one or several muscles, mainly observed at rest, but occasionally on posture. Myoclonus was always a rare event, superimposed on recordings of typical tremor, therefore we conclude that both are associated in these seven patients.

Myoclonus already had been reported in adult ET populations but was less common (<2% [4] *vs* 41% in our study). The frequency of myoclonus in our group of patients seems too important to consider a simple co-occurrence of symptoms (myoclonus being rare in children [22]). Given the fact that (i) all the patients fulfill diagnosis criteria of ET, (ii) polymyographic recording is concordant with ET and (iii) myoclonus is mild, we suggest that myoclonus constitutes an epiphenomenon associated to ET. Moreover, we have clearly demonstrated that there is no significant difference between patients with and without family history of ET regarding clinical especially tremor characteristics and regarding the presence or absence of myoclonus. Although the neurophysiological cortical markers were not studied, the polymyographic pattern of myoclonus (bursts duration and organization) were highly suggestive of a sub-cortical generator. The significance of myoclonus can only be hypothesized: it could be an under recognized accompanying motor sign in pediatric population or a maturational marker of ET in childhood, related to immature developing brain. It would be interesting to do a follow-up to evaluate the evolution in these patients. We suggest that identification of such myoclonus in a patient with tremor which fulfills the strict ET diagnostic criteria doesn’t jeopardize the diagnosis of COET.

Although our methodological approach does not allow to draw conclusion regarding the sex ratio, we noted that our small non selected group of childhood-onset ET patients is characterized by a 1:1 male/female ratio. In previous studies of COET, which enrolled patients diagnosed with ET between 1 and 3 decades ago, a clear male predominance has been described (60 to 74% of the patients are male) [7,10,12,13,14,15,16] (Table 4). It has been hypothesized that boys may manifest the disease younger than girls. Such ratio is not reported in patients with onset in adulthood: male and female are approximately equally affected [23,24]. Selection bias may explain the 1:1 ratio recorded in this series, especially given the short number of patients enrolled. However, our series is remarkable as it is the only one including patients with COET diagnosis established in recent years (after year 2011) and supported by neurophysiological recordings. It may be suggested that gender bias (girls are less likely to be referred), as commonly admitted in neurodevelopmental motor disorders including developmental coordination disorder [25,26], might also be involved in the overrepresentation of boys in previous historic series [7,12,13,14,15,16].

PedSQL assessment was decreased compared to healthy subjects especially in the self-report emotional scale: a significantly lower score was found for our patients compared to the reference cohort of 5961 healthy children [18] (64.58 ± 20.28 versus 78.21 ± 18.64 (p = 0.0114), the minimal clinically important difference given by PedsQL designers was 8.94); a trend was suggested between lower emotional subscore and (i) being a female or (ii) longer disease duration. No significant difference was found when comparing our patient’s score to patients with diabetes, obesity, cardiac condition, cancer, rheumatologic disease, gastrointestinal condition or end-stage renal disease as reported by Varni *et al* [27], although the PedsQL scores for our ET patients, both self-reported (79 ± 12.2) and parent/proxy reported (77.7 ± 17) were significantly better than in the cohort of cerebral palsy reported by Varni *et al* [27] (respectively 66.85 ± 16.73 (p = 0.0180) and 51.28 ± 18 (p < 0.001), minimal clinically important difference at 4.36).

Moreover, our study shows that all patients reported an impact of the tremor on their daily activities, 56% reported psychological distress, 31% underwent psychological follow-up, and 48% required personalized schooling help. QOL score of the COET children were comparable to those with diabetes, obesity, cardiac condition, cancer, rheumatologic disease, gastrointestinal condition or end-stage renal disease.

It is noteworthy that more than half of the patients/caregivers reported a worsening of the ET from onset, including extension to another body part, as suggested by Ghosh’s series [13].These findings emphasize the need for repeated and dedicated assessment of functional impairment related to ET in childhood; specific caution will be provided at adolescence and especially to girls; as the disorder is chronic, transition care will provide safe and smooth transfer of patients from pediatric to adult services [28].

Limitation deserves to be mentioned, especially the small number of patients that were enrolled. Some data extracted from medical files were occasionally missing. Some data were collected by familial interview, and therefore may be biased, especially concerning the course of the disease. To limit the possible bias related to multicentric recruitments and inter-investigator bias, we performed standardized clinical examination and formalized PMG recordings by a limited number of trained expert physicians.

This cross-sectional study provides a new perspective on clinical and electrophysiological characteristics of ET in childhood and warrants assessment of larger groups of children and longer follow-up.

## Additional File

The additional file for this article can be found as follows:

10.5334/tohm.803.s1 Supplementary tables. Tables 1 to 4.
