# Supplementary material for: Clinical and Electrophysiological Characterization of Essential Tremor in 18 Children and Adolescents
Source: Tremor Other Hyperkinet Mov (N Y). 2023 Dec 20;13:46. doi: 10.5334/tohm.803 (PMC10742103; doi:10.5334/tohm.803)
Supplement: Supplementary tables. — Tables 1 to 4. [file tohm-13-1-803-s1.pdf]

Supplementary table 1: exclusion criteria

| Exclusion criteria |
| --- |
| - abnormal neurological examination apart from tremor;<br>- family or personal history of neurological diseases such as Parkinson's disease or other degenerative parkinsonian syndrome, cerebellar ataxia, multiple sclerosis or WILSON's disease or personal history of stroke;<br>- family or personal history of dysthyroidism;<br>- delay in overall psychomotor or motor development;<br>- potentially tremor-inducing medication;<br>- known poisoning by heavy metals, bismuth, mercury, methyl bromide;<br>- sudden onset of tremor;<br>- suspicion of psychogenic tremor;<br>- brain MRI abnormalities, or the absence of brain MRI for a patient with no family history of essential tremor. |

Supplementary table 2: worsening and alleviating factors

| | At referral | At last follow-up |
|---|---|---|
| Worsening factors | 100% (16/16) | 93.3% (14/15) |
| More than two worsening factors | 31.3% (5/16) | 28.6% (4/14) |
| Emotion | 100% (12/12) | 92% (12/13) |
| Stress | 100% (15/15) | 100% (14/14) |
| Concentration | 9.1% (1/11) | 10% (1/10) |
| Fatigue | 75% (9/12) | 72.7% (8/11) |
| Caffeine | 0% (0/3) | 0% (0/4) |
| Infection | 11.1% (1/9) | 11.1% (1/9) |
| Fasting | 40% (4/10) | 27.3% (3/11) |
| Physical activity | 46.2% (6/13) | 58.3% (7/12) |
| Tobacco | 0% (0/2) | 0% (0/2) |
| Medication[1] | 11.1% (1/9) | 0% (0/9) |
| Others[2] | 11.1% (2/18) | 0% (0/18) |
| Alleviating factors | 11.1% (2/18) | 23.5% (4/17) |
| Concentration | 5.6% (1/18) | 5.9% (1/17) |
| Alcohol | 0% (0/5) | 20% (1/5) |
| Medication[3] | 11.1% (1/9) | 27.3% (3/11) |
| Others | 0% (0/18) | 0% (0/18) |

Qualitative data are presented as percentage (number of patients/number of patients with available data)

1: fluticason

2: prolonged writing (1); haste (1). The patients didn't know the effects of coffee, exciting substances, and tobacco on their tremor, or couldn't find a negative effect.

3: for all cases: propranolol

Supplementary table 3: Number of patients in the different subgroups of population

| | |
|---|---|
| Sex | Male: 9; female: 9 |
| Family history of tremor | Yes: 11; no: 7 |
| Age at onset | ≤6 years: 6; > 6 years: 12 |
| Age at inclusion | ≤13 years: 7; >13 years: 11 |
| Disease duration (from onset) | ≤5 years: 14; >5 years: 4 |

Supplementary table 4:

| | Presence of a family history of tremor | |
|---|---|---|
| | Yes | No |
| **Patients** | | |
| Number of patients | 11 | 7 |
| Age at onset (unknowm for one patient) | 6.91 ±3.67 | 5.83 ±3.49 |
| Age at assessment | 12.24 ±2.97 | 13.68 ±3.88 |
| Sex distribution (% of males) | 55 | 43 |
| **Tremor characteristics** | | |
| Course:<br>Progressive<br>Static<br>Improvement<br>Not known | <br>4<br>1<br>2<br>1 | <br>5<br>1<br>0<br>1 |
| Distribution at onset: bilateral upper limb | 100 (10/10) | 100 (7/7) |
| Distribution at last assessment: bilateral upper limb | 100 (9/9) | 100 (7/7) |
| **Polymyography** | | |
| Number of patients | 11 | 6 |
| Type of tremor:<br>Postural or kinetic (%)<br>Rest (%) | <br>91 (10/11)<br>0 (0/) | <br>83 (5/6)<br>17 (1/6) |
| Associated with myoclonus (%) | 45 (5/11) | 33(2/6) |
| Mean frequency of tremor (Hz) | 7.8 ±1.03 | 7.2 ±1.92 |
| Mean duration of bursts (ms) | 77.4 ±6.65 | 93.4 ±24.03 |
| Weight (500g) persistence (%) | 90 (9/10) | 100 (4/4) |
| Mean frequency for tremor persisting with 500 g weight (Hz) | 8.37±1.41 | 7.67 (n=3) ±1.25 |
| **Consequences** | | |
| Treatment proposition (%) | 40 (4/10) | 57 (4/7) |
| Fahn Tolosa Marin rating score | 25.62 ±13.80 | 26.60 ±11.89 |
| PedsQL score | 82.14 ±9.96 | 74.56 ±14.76 |

Qualitative data are presented as percentage (number of patients/number of patients with available data). Quantitative data are presented as mean ± standard deviation. PedsQL: Pediatric Quality of Life inventory.
